# Cognitive and social congruence in peer-assisted learning – A scoping review

**DOI:** 10.1371/journal.pone.0222224

**Published:** 2019-09-09

**Authors:** Teresa Loda, Rebecca Erschens, Hannah Loenneker, Katharina E. Keifenheim, Christoph Nikendei, Florian Junne, Stephan Zipfel, Anne Herrmann-Werner

**Affiliations:** 1 Department of Psychosomatic Medicine and Psychotherapy, University Hospital Tuebingen, Tuebingen, Germany; 2 Centre for Psychosocial Medicine, Department of General Internal Medicine and Psychosomatics, University Hospital Heidelberg, Heidelberg, Germany; Robert Bosch Krankenhaus, GERMANY

## Abstract

This scoping review presents an overview of cognitive and social congruence in peer assisted learning (PAL), as the positive effects of PAL have been shown to rely on these critical factors. The scoping review followed the guidelines of the preferred reporting items for systematic reviews and meta-analyses (PRISMA) statement. Databases were systematically searched for articles that focus on PAL and cognitive and social congruence. Participants of the studies included were medical, health science, polytechnic, law and paramedic students. Studies that assessed cognitive and social congruence by questionnaires with a 5-point Likert scale were regarded for meta-analytic pooling. Sixteen of 786 identified articles were included in the review, whereof 9 studies were considered for meta-analytic pooling. The meta-analytic pooling showed that tutees tend to see their student tutors as cognitively (*M*_*weighted*_ = 3.84; range of *M*_*weighted*_ *=* 2.69–4.56) and socially congruent (*M*_*weighted*_ = 3.95; range of *M*_*weighted*_ = 2.33–4.57). Further, characteristics of student tutors are summarized. This scoping review presents an overview and operationalization of cognitive and social congruence in PAL. Based on the presented meta-analytic pooling, cognitive and social congruence were found to represent relevant key factors in the PAL context. Thus, this theoretical background should be acknowledged as a core concept for tutorials within the medical curriculum.

## Introduction

Over the past decades, the concept of peer-assisted learning (PAL) has firmly established itself in the field of medical curricula [1–4]. Topping and Ehly [5] defined PAL as the “acquisition of knowledge and skills through active help and support among status equals or matched companions.” [5]. Peer-assisted learning has a long tradition in the teaching of students [6] and was used in numerous courses of study such as health sciences or law [7, 8]. Peer-assisted learning, further, plays a relevant role in the medical training [9–11] and is one of the most valuable teaching methods in the undergraduate medical education because all persons involved benefit from this learning concept [12]. So, PAL has been able to impact on the medical curriculum by enabling new ways of teaching where, rather than a professional teacher, peers are actively involved in the teaching process. Students acting as teachers are usually called “student tutors”, while the students being taught are characterized as “tutees”. In some studies, student tutors are also described by tutees as so-called journeymen or facilitators [13–15]. An overview of relevant terminology and definitions of PAL and its associated instructors can be found in Herrmann-Werner et al. [16].

Numerous studies have confirmed high levels of satisfaction with this instructional method, and tutees, in particular, have stressed the fact that they experienced themselves as being less anxious and receiving more honest, realistic and helpful feedback in a PAL context [9, 11, 13, 17–19].

### Findings of cognitive and social congruence in PAL

Cognitive congruence in the PAL context is described as student tutors and tutees sharing the same knowledge framework [7, 9, 13, 20, 21]. Student tutors have been shown to know where students are struggling and to be able to consider topics as important, difficult or as basic knowledge [9]. Furthermore, cognitive congruence is nurtured by the fact that student tutors use language that is familiar to tutees [7, 22–24]. All these factors enable student tutors to explain difficult topics or concepts at appropriate levels for tutees’ comprehension [7, 17, 20, 21].

Social congruence in PAL is created by the fact that student tutors and tutees share similar social roles [7, 9, 25–27]. Consequently, tutees feel more comfortable with their student tutors than with their teachers [7, 9, 26, 27]. Social congruence enables student tutors to be more supportive and empathic towards tutees’ needs, difficulties, and expectations and to communicate in informal way [7, 9, 15]. Furthermore, student tutors demonstrate social congruence by being interested in tutees’ academic workloads and daily lives because they themselves have already completed the same course successfully at an earlier stage of their studies [7, 9, 15]. Several studies have postulated that social congruence might also be reflected by student tutors encouraging their tutees to actively participate in class by giving feedback, taking risks and asking questions [13, 18, 28].

### Cognitive and social congruence as key factors for effective PAL

Past studies have indicated that the effectiveness of PAL seemed to be rooted in the concepts of cognitive and social congruence [7, 9, 10, 23, 29]. Several studies postulated that cognitive and social congruence between student tutors and tutees could result in a powerful PALexperience [7, 9, 13, 20]. Huhn et al. [30] showed that cognitive and social congruence also presented key factors for success in regard to the education of international medical students.

The concept of cognitive and social congruence was first investigated by Schmidt and Moust [7] as a theoretical model of student tutor performance in an actual teaching session. Their theoretical model linked subject-matter expertise, social congruence and cognitive congruence to the functioning of small student groups, time spent on studying individually and student outcomes such as academic success [7, 31]. The theoretical model showed that higher social congruence and higher subject-matter expertise could contribute to higher cognitive congruence among student tutors [7, 32]. In turn, higher cognitive congruence might lead to better functioning of small student groups and greater performance, expressed by higher intrinsic motivation, more time spent on individual study and better examination results [7, 32]. In addition, Lockspeiser et al. [9] looked at the two concepts of cognitive and social congruence in a medical program with second-year medical students serving as near-peer student tutors for first-year students. First-year students involved in this study reported that their student tutors were able to anticipate the problems they had when understanding new concepts in class and that they automatically shared their experiences and strategies, thereby assisting the first-year students to overcome their learning difficulties [9, 31]. Further studies about cognitive and social congruence in a peer-teaching context attempted to identify the optimal distance along a “peer-teaching spectrum” in regard to the matching of cognitive and social congruence between student tutors and tutees by comparing the assessment of tutees when taught by student tutors, staff tutors or junior doctors [11, 12, 28].

### Aim of the investigation

Despite the widely accepted fact that cognitive and social congruence might represent highly relevant concepts in the success story of PAL, these two terms have not yet been clearly defined and operationalized. Only Schmidt and Moust [7, p. 710] stated possible definitions as cognitive congruence ‘referred to a student tutor’ ability to use explanations that were easily grasped by students’ and social congruence ‘referred to a student tutor’s willingness to act informally with students and displayed a caring attitude’. This scoping review aims to, generally, define and operationalize cognitive and social congruence based on previous findings like Schmidt and Moust [7]. To the best of our knowledge, there has been no scoping review thus far of these crucial factors in PAL.

Further, since the results of previous studies are very heterogeneous, this paper purposes to present an overview of how cognitive and social congruence on the part of student tutors are perceived by tutees. When possible, data were used for an international comparison in a meta-analytic pooling. In summary, the following research questions will be addressed in this scoping review:

1. How is cognitive and social congruence, generally, defined and operationalized in peer tutoring?
2. How cognitively and socially congruent (based on self-reported instruments) were the student tutors perceived by their tutees in previous studies?
3. What further characteristics/competencies should student tutors have in order to be cognitively and socially congruent with their students?

## Materials and methods

The present study is based on the Preferred Reporting Items for Systematic Reviews and Meta-Analyses (PRISMA) statement [33, 34].

### Ethical considerations

The study received ethical approval from the Ethics Committee of Tuebingen Medical Faculty (No. 129/2017BO2) in April 2017.

### Protocol

No review protocol exists.

### Study search

Relevant studies were searched in an electronic general database (Google Scholar), in two databases with medical backgrounds (PubMed, PsycINFO) and two databases with educational scientific backgrounds (ERIC, FIS). The search terms used for each electronic database included the following: "cognitive"[All Fields]) OR social[All Fields]) AND congruence[All Fields]) OR (peer[All Fields] AND assisted[All Fields] AND ("learning"[MeSH Terms] OR "learning"[All Fields]))) OR (near[All Fields] AND peer[All Fields] AND ("learning"[MeSH Terms] OR "learning"[All Fields]))) OR (near[All Fields] AND peer[All Fields] AND ("education"[Subheading] OR "education"[All Fields] OR "teaching"[All Fields] OR "teaching"[MeSH Terms]))) OR (peer[All Fields] AND tutoring[All Fields])) OR ("students, medical"[MeSH Terms] OR ("students"[All Fields] AND "medical"[All Fields]) OR "medical students"[All Fields] OR ("medical"[All Fields] AND "students"[All Fields]))) AND ("students"[MeSH Terms) OR "students"[All Fields]). Additionally, relevant literature associated with this topic was searched for in grey literature using Open Grey. From November 2016 until February 2018, we searched for relevant studies published between 1993 and February 2018. The search was started in 1993 because Moust [35] was one of the first to mention cognitive and social congruence in peer learning.

### Study selection

For inclusion in the scoping review, studies needed to fulfil the following criteria that were oriented on the PICO (Population, Intervention, Comparison/Control and Outcome) approach [33]. Cross-references of the studies included were also checked. (1) Studies from every discipline were included due to the limited number of studies found. (2) The students must have been taught by student tutors, who had to be more academically experienced than the tutees in the taught field. (3) The extent of cognitive and social congruence was considered from the tutees’ point of view through Likert-scale based instruments used in the reported studies. (4) Tutees had to evaluate the levels of cognitive and social congruence of their student tutors … Finally, studies that had used staff tutors, junior doctors or specialists as peer teachers were excluded from the present investigation.

### Study design

Studies with cross-sectional or longitudinal designs were included in the present study. Reviews, comments, editorials, case studies or letters to the editor were excluded. Relevant studies should to have been peer-reviewed (except grey literature) and written in either the English or the German language.

### Study outcomes

The study outcomes had to focus on cognitive and social congruence and associated factors on a behavioral level demonstrated by student tutors and assessed by their tutees.

### Screening and selection of studies

Two authors (HL and TL) independently screened titles and abstracts of publications focusing on cognitive and social congruence in line with the inclusion criteria. According to the screening process, the articles were categorized as “eligible” or “ineligible” studies. Studies found to be ineligible by both reviewers were excluded immediately. Those that at least one reviewer judged eligible were included. Cohen’s kappa was calculated to test for inter-rater reliability. The studies first declared eligible were screened by full-text analysis by the reviewers (HL and TL). Finally, all articles that were declared eligible based on full-text analyses were included. Whenever the two reviewers disagreed, the corresponding article was screened by a third reviewer (JT), who then decided to include or exclude the article.

### Quality assessment of included studies

For quality assessment, the study extracted the following dimensions: study design, participants, population size, applied instruments and reported results of cognitive and social congruence. All included studies were chosen with regard to their focus on the investigation of cognitive and social congruence and corresponding primary descriptive data. Cognitive and social congruence had to be measured by questionnaires that assess cognitive and social congruence such as the self-report questionnaire adapted by Schmidt and Moust [7] or by items that have been shown to be associated with cognitive and social congruence. The questionnaire of Schmidt and Moust [7] was shown to be reliable and valid [31]. To prevent the risk of bias in studies with associated items, we checked the single items carefully by study if they were associated with cognitive and social congruence. Cognitive congruence, for example, was associated with giving helpful feedback, while social congruence might reduce anxiety in tutees [19]. Further, cognitive congruence was measured by items such as “The student tutor asked questions we could understand”. Social congruence, for example, was represented by items such as “The student tutor showed interest in our personal lives”.

### Measurement of cognitive and social congruence

Half of the included studies used the Tutor Evaluation Questionnaire developed by Schmidt and Moust [7] to measure cognitive and social congruence. The questionnaire consists of 10 items on a 5-point Likert scale (first version only consists of 3-point Likert scale) from “fully disagree” to “fully agree” or from “not true at all” to “very true”. Several studies tested the reliability and validity of the questionnaire by calculating Hancock’s coefficient ranging from 0.70 (for social congruence) to 0.77 (for cognitive congruence) [14, 18, 31, 36, 37]. The further questionnaires used to assess cognitive and social congruence also proved to be reliable and valid with Cronbach's alpha from 0.80 to 0.87 [8, 11, 12, 38].

### Statistical preparation of meta-analytic pooling

For meta-analytic pooling, we calculated the weighted mean values of the measurement of cognitive and social congruence based on the size of the sample and the results of the studies included. Mean values were used for meta-analytic pooling because only mean values of the instruments were reported. Whenever the resulting mean values of cognitive and social congruence were not reported in the articles, we contacted the authors to ask for the raw data. When studies reported only single items concerning cognitive and social congruence or results from several classes, we calculated the overall mean values of cognitive and social congruence based on the reported values. If necessary, values were recalculated and adapted to a 5-point Likert scale. As mentioned above, only mean values of student tutors (e.g. senior medical students) are presented in this study. All calculations were made by using IBM SPSS V24.

## Results

### Search results and quality assessment

**Fig 1:**
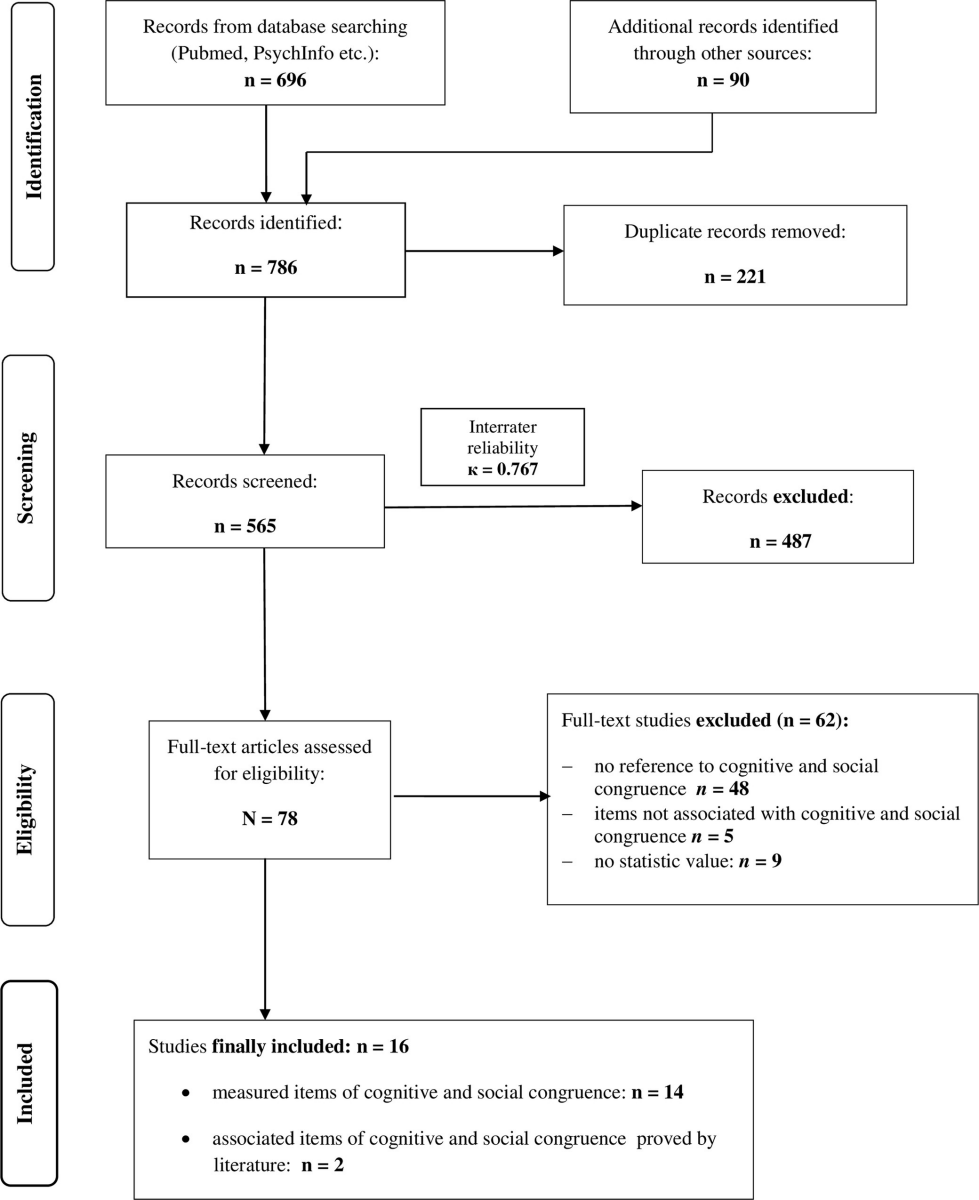
Flowchart of found studies.

According to the PRISMA statement, the flowchart (Fig 1) presents the search results and the associated quality assessment adapted for the aim of this review. Overall, 786 records were identified by searching databases. After removing duplicates and screening titles as well as abstracts, 78 articles remained to be evaluated by full-text reading to determine their eligibility. In total, 62 articles were excluded after full-text analysis: 48 articles had no reference to cognitive and social congruence, 9 presented no statistical values, and 5 demonstrated items that were associated with behavioral patterns in PAL but not directly with cognitive and social congruence. The inter-rater reliability was substantial, with Cohen’s kappa κ = 0.767 (*p* < .001).

Finally, 16 studies were included in the review. We differentiated between investigations that assessed cognitive and social congruence directly by using questionnaires (n = 14) and studies that measured these two constructs by associated items presented in former studies (n = 2) (see Table 1). The participants of the included studies were medical, health science, polytechnic, law and paramedic students.

**Table 1:** Overview of studies investigating cognitive (CC) and social congruence (SC).

Studies of questionnaires that measure cognitive and social congruence				
Authors	Country	Instruments	Participants	Results of CC and SC
Chng et al.[36]^a^	Singapore	Self-report questionnaire adapted from Schmidt and Moust [7] (5-point Likert scale, 10 items)	Students: N = 223 Student tutors: N = 7	M(CC) = 3.65 (0.20) M(SC) = 3.27 (0.37)
Chng et al. [18]^a^	Singapore	Self-report questionnaire adapted from Schmidt and Moust [7] (5-point Likert scale, 10 items)	Students N = 77 Student tutors N = 11	Study 2: M(CC) = 3.94 (0.19) M(SC) = 3.83 (0.28)
Rotgans and Schmidt [14]	Singapore	Tutor Evaluation Survey developed by Schmidt and Moust [7] (5-point Likert scale, 10 items)	Students N = 498 Student tutors: not reported	M(CC) and M(SC) are not reported secondary outcome: Path models: SC bears CC with ß = 0.28;
Schmidt and Moust [7] ^a^	Netherlands	Tutor Evaluation Survey developed by Schmidt and Moust [7] (3-point Likert scale, 10 items)	Students: N = 1452 Student tutors: N = 261	M(CC) = 2.69 (0.447) M(SC) = 2.74 (0.371) *Overall M (CC) = 4*.*48* ^*b*^ *Overall M (SC) = 4*.*57*^*b*^
Williams [31] (Chapter 4)	Singapore	Tutor Evaluation Survey developed by Schmidt and Moust [7] (5-point Likert scale, 10 items)	Students: N = 10854 Student tutors: N = 268	M(CC) and M(SC) are not reported secondary outcome: Path model: SC bears CC with ß = 0.51
Williams et al.[37] ^a^	Singapore	Tutor Evaluation Survey developed by Schmidt and Moust [7] (5-point Likert scale, 10 items)	Students: N = 16047 Student tutors: N = 762	Semester 2009–1: M(CC) = 3.79 M(SC) = 3.76 Semester 2009–2 M(CC) = 3.78 M(SC) = 3.75 Semester 2010–1 M(CC) = 3.80 M(SC) = 3.77 *Overall M (CC) = 3*.*79*^*b*^ *Overall M (SC) = 3*.*76*^*b*^
Williams [31] (Chapter 5)	Singapore	Tutor Evaluation Survey developed by Schmidt and Moust [7] (5-point Likert scale)	Students: N = 12108 Student tutors: N = 376	M(CC) and M(SC) are not reported secondary outcome: Student tutors with qualifications or experience are rated higher for CC
Yew and Yong [15]^a^	Singapore	Tutor Evaluation Survey developed by Schmidt and Moust [7] (5-point Likert scale, 10 items)	Students: N = 12358 Student tutors: N = 1065	M(CC) = 3.84 (0.25) M(SC) = 3.77 (0.35)
De Rijdt et al. [38]^a^	Netherlands	Online questionnaire with 12 items (5-point Likert scale) including four factors: stimulating function, cognitive congruence, social congruence and expertise	Students: N = 751 Student tutors: N = 23	Course A: M(CC) = 3.8 (1.0) M(SC) = 4.1 (0.9) Course B: M(CC) = 3.9 (1.1) M(SC) = 4.1 (1.1) Course C: M(CC) = 4.0 (0.7) M(SC) = 4.1 (0.7) Course D: M(CC) = 4.2 (0.7) M(SC) = 4.3 (0.7) *Overall M(CC) = 3*.*96*^*b*^ *Overall M(SC) = 4*.*15*^*b*^
Moust and Schmidt [8]^a^	Netherlands	Questionnaire with 39 items (5- point Likert scale) including 6 categories: use of expertise, cognitive congruence, test orientation, authority, role congruence, cooperation orientation	Students: N = 352 Student tutors: N = 11	First course: M(CC) = 3.7 (0.4) M(Role Congruence) = 3.0 (0.6) M(Authority) = 1.7 (0.6) Second course: M(CC) = 3.6 (0.5) M(Role Congruence) = 3.1 (0.6) Authority: M(Authority) = 1.5 (0.4) *Overall M(CC) = 3*.*65*^*b*^ *Overall M(SC) = 2*.*33*^*b*^ *because Role Congruence and Authority can be merged into social congruence[7]*
Hall et al. [10]	Southampton, UK	Formal evaluation form (4-point Likert scale) with - items relating to CC were use of time and relevance - items relating to SC were enjoyment	Students N = 60 Student tutors: N = 10	M(CC) and M(SC) are not reported (only in graphic)
Hall et al. [11]	Southampton, UK	Questionnaire (5-point Likert scale) with - items relating to CC were use of time, delivery and relevance of content - items relating to SC were enjoyment and approachability	Students N = 98 Student tutors N = 5	M(CC) and M(SC) are not reported (only in graphic) secondary outcome: Enjoyment as criteria of SC and use of time as well as delivery of teaching as criteria for CC scored significantly higher for senior medical students compared to junior doctors
Stephens et al. [12]^a^	Southampton, UK	Questionnaire (5-point Likert scale) with - items relating to CC were Use of time, delivery and relevance of content - items relating to SC were enjoyment and approachability	Students: N = 492 Student tutors: N = N. A.	CC: M (Relevance) = 4.59 M (Delivery) = 4.20 M (Use of Time) = 4.19 SC: M(Approachability) = 4.60 M(Enjoyment) = 3.96 *Overall Mean CC*: *4*.*56*^*b*^ *Overall Mean SC*: *4*.*28*^*b*^
Tayler et al. [28]	Southampton, UK	Questionnaire (5-point Likert scale) with 15 items: - items relating to CC were Use of time, delivery and relevance of content - items relating to SC were enjoyment and approachability	Students: N = 240 Student tutors: N = 119	M(CC) and M(SC) are not reported (only in graphic) secondary outcome: Approachability of teacher, teacher’s receptiveness to student input, awareness of learning outcomes and teacher’s investment in examination success scored significantly higher for near-peer teachers compared to registrar or consultant tutors
**Studies with single items associated with cognitive and social congruence**				
Authors	Country	Instruments	Participants	Results of CC and SC
Lockspeiser et al. [9]^a^	San Francisco, California	Questionnaire with 14 items (5- point Likert scale)	Students: N = 111 Student tutors: N = N. A.	Mean of items that might be associated with CC = 3.8 (0.54) Mean of items that might be associated with SC = 3.55 (0.80)
Williams and Nguyen [19]^a^	Victoria, Australia	Clinical Teaching Preference Questionnaire CTPQ (5-point Likert-Scale, 11 items)	Students: N = 86 Student tutors: N = 25	CC: M(more honest, realistic and helpful feedback) = 2.69 (1.08) SC: M(less anxious) = 2.71 (1.02)
**Weighted Mean of CC = 3.84** (range of *M* = 2.69–4.56) **Weighted Mean of SC = 3.95** (range of *M* = 2.33–4.57)				

^a^Studies included in the meta-analytic pooling

^b^ Mean values are self-calculated based on the results of the study (adapted to 5-point Likert scale, different semester or courses, single items)

### Meta-analytic pooling

In the meta-analytic pooling, 10 of 16 studies fulfilled the inclusion criteria and were thereby included; for 5 of these 10 studies, we calculated the mean value ourselves, as only single items measuring cognitive and social congruence or results from other Likert-scale or from several classes were reported in the original versions of these studies. We considered the mean values of cognitive and social congruence assessed on a 5-point Likert scale.

**Fig 2:**
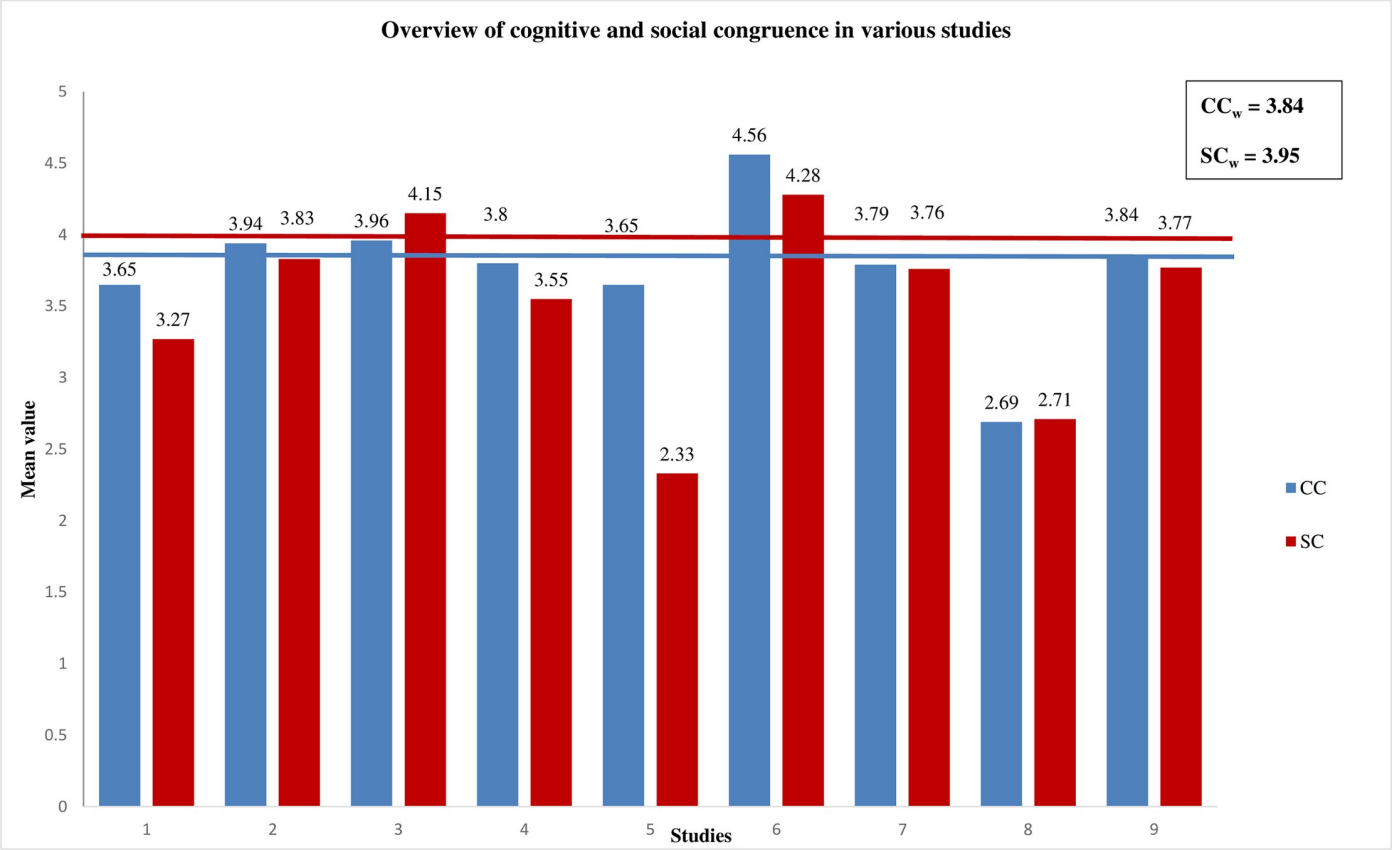
Overview of mean value of cognitive (CC) and social congruence (SC) in various studies.

Regarding all studies of the meta-analytic pooling that measured cognitive and social congruence on a 5-point Likert scale (from “fully disagree” to “fully agree”), the weighted mean value of cognitive congruence was *M* = 3.84 (range of *M* = 2.69–4.56). The weighted mean value of social congruence was *M* = 3.95 (range of *M* = 2.33–4.57) (see Table 1). The sample sizes of the included studies ranged from N = 77 to N = 12,358. When the studies were separated into two groups, including cognitive and social congruence assessed by self-reported questionnaires or measured by associated items, the weighted mean values differed slightly in cognitive and social congruence between the two groups. The weighted mean value of cognitive congruence was lower for the studies with associated items (*M* = 3.32; range of *M =* 2.69–3.80) when compared to the weighted mean value of cognitive congruence for the studies with valid and reliable questionnaires (*M* = 3.98; range of *M =* 3.65–4.56). Furthermore, we found similar results for social congruence when including both groups of studies. The weighted mean value of social congruence was *M* = 3.18 (range of *M =* 2.71–3.55) for studies with associated items and *M* = 3.75 (range of *M =* 2.33–4.57) for studies with assessed questionnaires. Overall, the results show that tutees seem to consider their student tutors as cognitively and socially congruent, respectively (see Fig 2).

### Further significant results on cognitive and social congruence

Several studies from a working group in Southampton (UK) investigated the optimal distance along the peer-teaching spectrum in terms of students’ perceived distance from their teachers on such a range. The students were asked to rate their perceived cognitive and social congruence to a senior medical student tutor vs. a junior doctor tutor, faculty tutor or consultant tutor [11, 12, 28]. Student tutors scored significantly higher when looking at criteria for social congruence (“enjoyment” and “approachability”) as well as for cognitive congruence (“relevance”, “use of time” and “delivery of teaching”) [28]. Consequently, student tutors were perceived as more cognitively and socially congruent by tutees compared to faculty or consultant tutors in a peer-learning context [11, 12, 28].

Other studies investigated cognitive and social congruence as teacher characteristics or teaching behaviors within different path models to find dependencies of different variables associated with PAL such as “tutorial group functioning”, “situational interests” or “study achievement” [7, 14, 31]. In every path model calculated, the results showed that cognitive congruence is influenced by social congruence with ß ranging from 0.28 to 0.51 and by subject-matter expertise (ß = 0.41–0.58). In addition to social congruence, expertise presented a relevant ascendant for cognitive congruence. When regarding further PAL factors e.g. tutorial group functioning, Schmidt and Moust [7] demonstrated in their path model that cognitive and social congruence might also facilitate group performance in a direct causal way (CC: ß = 0.18; SC: ß = 0.24).

Williams et al. [37] conducted a study with 762 student tutors who were rated by 16,047 students focusing on the variability and variance components of cognitive and social congruence over three semesters, focusing on differences between student tutors and semesters. Here, social congruence showed the highest variance with 60.5% of difference between the student tutors followed by cognitive congruence with 56.1% variance that differed between student tutors. Only variance with 0.1% of difference was found between the semesters. These results indicate that cognitive and social congruence varies between student tutors but not between semesters.

In a further investigation, Williams [31] aimed to identify factors that might affect student tutors’ behaviors including cognitive and social congruence. Using ANOVA, they demonstrated that the factors “possession of educational qualification” (*p* < .01) and “year of experience” (*p* < .05) had an impact on cognitive congruence. Student tutors who possessed an educational qualification or were more experienced were rated higher for cognitive congruence by students. Social congruence, however, wasn’t influenced by any factor like gender or age (*p* > .05).

## Discussion

This study aimed to present a scoping review of cognitive and social congruence in peer learning, including a meta-analytic pooling of previous studies on this topic. In the meta-analytic pooling, mean values of cognitive and social congruence tended to the direction of nearly “agree”, indicating that students might perceive their student tutors as cognitively and socially congruent. We synthesized the results of previous investigations of cognitive and social congruence.

### Cognitive and social congruence as operational key factors

As the results from the reported studies showed, cognitive and social congruence could be identified as important key factors in the peer-learning context. The weighted mean value calculated for cognitive congruence could validate that tutees tend to perceived their student tutors as cognitively congruent. Further, student tutors were perceived as more honest, realistic and helpful due to greater cognitive congruence between tutee and student tutor[19]. Moreover, greater cognitive congruence might imply that student tutors could better understand the problems that tutees might experience [7, 9]. Overall, student tutors are seen as more cognitively congruent than faculty tutors [9, 38].

The weighted mean value calculated for social congruence indicated that tutees also seemed to see their student tutors as socially congruent. This result of social congruence might be interpreted best in terms of the relevance of creating an informal means of communication and relationship-building with the tutees by the student tutor [7, 20, 36]. Chng et al. [36] stressed the importance of a student tutor’s willingness to establish an informal relationship with tutees and to demonstrate an attitude of genuine interest in them. Expressing interest in the tutees and their lives has been shown to have the greatest impact on the progress made by tutees in a peer-learning context [36]. Overall, tutees feel more comfortable when taught by student tutors [7, 9, 26]. The student tutors are perceived to behave more supportively and empathically towards tutees’ needs, difficulties and expectations [7, 9, 15].

### Characteristics of student tutors

Several studies reported that student tutors were evaluated more cognitively and socially congruent by tutees in comparison to faculty or consultant tutors [11, 12, 28]. Therefore, student tutor should be similar in age and level of medical training like tutees but should have more expertise in the taught topic. Schmidt and Moust [7] supported this statement by showing that social congruence as teacher characteristic and expertise were a relevant ascendant for cognitive congruence.

### Discrepancies of cognitive and social congruence

Although there were apparent discrepancies between the mean values of the studies included for both concepts (ranging from 2.33 to 4.56), the relevance of cognitive and social congruence in PAL has been supported by various studies previously [11, 12, 20, 28]. The observed discrepancies in the present investigation might best be explained through studies aiming to find the optimal distance along the peer-teaching spectrum in terms of tutees’ perceptions of cognitive and social congruence [11, 12, 28]. Stephens et al. [12] supported this idea by presenting significant differences between fourth- and fifth-year medical students in the feedback criteria of cognitive and social congruence when teaching second-year medical students. The results showed lower scores for feedback criteria for cognitive and social congruence for the fifth-year medical students when compared to the fourth-year medical students [12]. However, when comparing the feedback criteria results of cognitive and social congruence between third- and fourth-year medical students, no significant differences were found. Moreover, the scores of feedback criteria differed significantly when comparing the results from the fifth-year medical students with the results from junior doctors.

### Reflection of cognitive and social congruence in the literature

Despite the detailed and elaborated investigation of PAL addressed by many studies, few studies have focused on the concept of cognitive and social congruence in this learning context. Most of the previous studies investigated, in particular, the effectiveness of PAL or tutorials led by student tutors [26]. Therefore, cognitive and social congruence arise solely as presumed phenomena of effectiveness but are not regarded more closely on a behavioral level [20, 26, 39, 40].

The studies included in this review fulfilled the quality assessment for quantitative studies and assessed cognitive and social congruence through the use of valid and reliable questionnaires. Furthermore, they reported mutual dependencies of cognitive and social congruence with outcomes of the PAL context such as academic achievements of the students and student tutor effectiveness [18, 31, 36]. However, they didn’t consider cognitive and social congruence more closely on a behavioral level of student tutors and tutees. Moreover, they reported their outcomes of cognitive and social congruence in very different ways; therefore, it was difficult to provide a good overview.

This study presents the first scoping review of cognitive and social congruence in peer learning. Moreover, appropriate data were included in a meta-analytic pooling, and relevant factors associated with cognitive and social congruence such as tutorial group functioning were summarized.

One limitation of the present analysis was the missing consideration and evaluation of subject-matter expertise in the scoping review of cognitive and social congruence. In many studies, expertise was investigated together with cognitive and social congruence in the peer-learning setting [7, 14, 31, 36, 37]. The present investigation, however, focused on cognitive and social congruence because expertise was assessed solely by two items within the Tutor Evaluation Survey by Schmidt and Moust [7]. Furthermore, De Rijdt et al. [38] suggested that expertise might play a less relevant role, as the authors found that students did not miss a tutor’s expertise when it was compensated by cognitive congruence. Additionally, this review only focused on student tutors who were more advanced than the tutees and not on same-level PAL.

Future studies could focus on the relationship between cognitive and social congruence because several investigations resulted in the conclusion that social congruence presented an ascendant for cognitive congruence when regarding path models [7, 14, 31]. Furthermore, cognitive and social congruence influenced other PAL factors such as group performance or study motivation in a direct and separate way [7]. Hence, future studies should focus on identifying relevant factors of cognitive and social congruence on a behavioral level in order to derive specific measures and recommendations for action. Here, the teaching of student tutors should be compared to teaching of non-peer students like faculty tutors in order to receive various insights of PAL. In summary, this scoping review aims to present an overview and operationalization of cognitive and social congruence in PAL. It strengthens the relevance of the student tutor and tutee being cognitively and socially congruent for effective peer learning. Cognitive congruence implies that student tutor and tutee share a similar knowledge framework and use a familiar language [7, 9]. Social congruence is represented by the student tutor and tutee sharing similar social roles and an informal style of communication [7, 9, 20, 26, 36]. Future studies might investigate the relationship between cognitive and social congruence and associated factors like group performance or study motivation in the PAL context.

## Supporting information

S1 PRISMA Checklist (DOCX)
